# Acknowledgement

**DOI:** 10.1093/texcom/tgad006

**Published:** 2023-05-06

The Editor of *Cerebral Cortex Communications* would like to thank the following reviewers who have helped us from January 1 to December 31 of 2022.

Adamaszek, Michael

Ayata, Cenk

Barcelo, Francisco

Brooks, David J

Chan, Annie

Cooper, Robert J

Cooper, Samuel

D'Arcy, Ryan C.N.

de Blank, Peter

Dell'Acqua, Roberto

Ebner, Timothy

Elison, Jed

Fedorenko, Evelina

Figueroa, Johnny

Garcia-Garcia, Isabel

Gruskin, David

Hahn, Lukas

Herrmann, Martin J

Herrojo Ruiz, María

Iachini, Tina

Kartha, Arathy

Keitel, Anne

Kim, Hyun K

Kim, Jae

Kim, Seong-Gi

King, Andrew

Kuceyeski, Amy

Li, Lin

Lovato, Irene

Lunghi, Claudia

Maekawa, Fumihiko

Martinez-Lincoln, Amanda

McCabe, Brian

Meng, Chun

Miller, Jillian

Mohan, Krithika

Nandy, Rajesh

Naud, Richard

Niemeier, Matthias

Nomura, Taishin

Oleskiw, Timothy

Ono, Kentaro

Orio, Patricio

Pavlova, Marina

Perrey, Stephane

Picard, Nathalie

Pollonini, Luca

Richards, Todd

Rousselet, Guillaume

Salmitaival, Juha

Scheinost, Dustin

Schmidlin, Eric

Schreiner, Christoph

Scrivener, Catriona

Sheinberg, David

Shine, James

Shirazi, Seyed Yahya

Shoemaker, J Kevin

Sokolov, Arseny

Stauffer, William R

Steel, Adam

Strauss, Antje

Tamura, Keita

van Ede, Freek

Vanduffel, Wim

Verberne, Tony

Wang, Xingchao

Whitwell, Robert

Yap, Pew-Thian

Yaramothu, Chang

Yuan, Zhen

